# Effect of Tumor Necrosis Factor-Alpha on Erythropoietin- and Erythropoietin Receptor-Induced Erythroid Progenitor Cell Proliferation in β-Thalassemia/Hemoglobin E Patients

**DOI:** 10.4274/tjh.2014.0079

**Published:** 2015-12-03

**Authors:** Dalina I Tanyong, Prapaporn Panichob, Wasinee Kheansaard, Suthat Fucharoen

**Affiliations:** 1 Mahidol University Faculty of Medical Technology, Department of Clinical Microscopy, Nakhon Pathom, Thailand; 2 Mahidol University Thalassemia Research Center, Institute of Molecular Biosciences, Nakhon Pathom, Thailand

**Keywords:** Erythropoietin, β-Thalassemia/hemoglobin E, apoptosis

## Abstract

**Objective:**

Thalassemia is one of the genetic diseases that cause anemia and ineffective erythropoiesis. Increased levels of several inflammatory cytokines have been reported in β-thalassemia and might contribute to ineffective erythropoiesis. However, the mechanism by which tumor necrosis factor-alpha (TNF-α) is involved in ineffective erythropoiesis in thalassemic patients remains unclear. The objective of this study is to investigate the effect of TNF-α on the erythropoietin (EPO) and erythropoietin receptor (EPOR) expression involved in proliferation of β-thalassemia/hemoglobin (Hb) E erythroid progenitor cells compared with cells from healthy subjects.

**Materials and Methods:**

CD34-positive cells were isolated from heparinized blood by using the EasySep® CD34 selection kit. Cells were then cultured with suitable culture medium in various concentrations of EPO for 14 days. The effect of TNF-α on percent cell viability was analyzed by trypan blue staining. In addition, the percentage of apoptosis and levels of EPOR protein were measured by flow cytometry.

**Results:**

Upon EPO treatment, a higher cell number was observed for erythroid progenitor cells from both healthy participants and β-thalassemia/Hb E patients. However, a reduction of apoptosis was found in EPO-treated cells especially for β-thalassemia/Hb E patients. Interestingly, TNF-α caused higher levels of cell apoptosis and lower levels of EPOR protein in thalassemic erythroid progenitor cells.

**Conclusion:**

TNF-α caused a reduction in the level of EPOR protein and EPO-induced erythroid progenitor cell proliferation. It is possible that TNF-α could be involved in the mechanism of ineffective erythropoiesis in β-thalassemia/Hb E patients.

## Abstract (tr)

**Amaç:**

Talasemi anemi ve inefektif eritropoeze neden olan genetik hastalıklardan birisidir. Enflamatuvar sitokinlerin bir çoğunun seviyelerinde artma b-talasemide gösterilmiş olup, bu durum inefektif eritropoeze katkıda bulunabilir. Ancak, tümör nekrozlaştırıcı faktör-alfa’nın (TNF-α) talasemik hastalarda inefektif eritropoeze nasıl bir mekanizma ile neden olduğu bilinmemektedir. Bu çalışmanın amacı b-talasemi/hemoglobin (Hb) E eritroid öncül hücrelerinde sağlıklı kontrollerin hücreleri ile karşılaştırıldığında TNF-α’nın eritropoetin (EPO) ve eritropoetin reseptör (EPOR) sunumu üzerine etkisinin araştırılmasıdır.

**Gereç ve Yöntemler:**

CD34-pozitif hücreler EasySep® CD34 seçim kiti yardımı ile heparinli kandan izole edildi. Hücreler 14 gün boyunca uygun kültür ortamında değişik EPO konsantrasyonlarında kültürde bekletildi. TNF-α’nın hücre canlılık yüzdesine etkisi tripan mavisi boyası ile incelendi. Bunun yanında, apopitoz yüzdesi ve EPOR protein seviyeleri akış sitometrisi ile ölçüldü.

**Bulgular:**

EPO tedavisi ile eritroid öncül hücrelerinin sayısında hem sağlıklı katılımcılarda hem de b-talasemi/Hb E hastalarında artış olduğu görüldü. Ancak özellikle b-talasemi/Hb E hastalarında EPO ile muamele edilmiş hücrelerde apopitozda azalma görüldü. İlginç olarak, TNF-α talasemik eritroid öncül hücrelerde hücre apopitoz oranında artmaya ve EPOR protein seviyelerinde azalmaya neden oldu.

**Sonuç:**

TNF-α EPOR protein düzeyi ve EPO ile uyarılmış eritroid öncül hücre çoğalmasında azalmaya neden oldu. b-talasemia/Hb E hastalarında TNF-α inefektif eritropoez mekanizmasında yer alıyor olabilir.

## INTRODUCTION

Thalassemia is a genetic disease. The major pathophysiological features include ineffective erythropoiesis and anemia. In terms of ineffective erythropoiesis, the mechanism includes increased intramedullary erythroid death and arrested proliferation of erythroid progenitors, which plays an important role in β-thalassemia [1]. β-Thalassemia/hemoglobin (Hb) E is the commonest form in many Asian countries. In Thailand, the World Health Organization estimates that at least 100,000 new cases of the disease will be seen in the next few decades. The pathophysiology is more complex and the cause of the variability of the severity remains unknown [2].

Erythropoietin (EPO) is a glycoprotein hormone required for the survival, proliferation, and differentiation of committed erythroid progenitor cells. The erythropoietin receptor (EPOR) belongs to the cytokine receptor superfamily, which includes receptors for other hematopoietic growth factors such as interleukins, colony-stimulating factors, and growth hormone. EPO binds to EPOR and causes the signaling pathways to control survival and proliferation of erythroid cells [3]. Survival signaling by EPOR is essential for erythropoiesis and for its acceleration in hypoxic stress. Several apparently redundant EPOR survival pathways were identified in vitro, raising the possibility of their functional specialization in vivo [4].

One of the most important pathophysiologies of β-thalassemia is ineffective erythropoiesis. Inflammatory cytokines such as tumor necrosis factor-alpha (TNF-a) were reported to inhibit erythropoiesis in vivo and vitro [5]. TNF-a induces an increase of apoptosis within the compartments of immature erythroblasts and a decrease in mature erythroblasts. However, the exact mechanism remains unclear.

The objectives of this study were to study the effect of TNF-a on EPO and EPOR protein involved in proliferation of erythroid progenitor cells in β-thalassemia/Hb E patients.

## MATERIALS AND METHODS

### Blood Samples

Heparinized blood samples were collected from 5 healthy subjects and 5 β-thalassemia/Hb E patients. The thalassemia patients in this study had the moderate to severe type of the disease. They were transfusion-dependent and splenectomized. However, patients had no transfusions or iron chelation at least 3 weeks before the time of sampling. Diagnosis of thalassemia was based on family history, red cell indices, and hemoglobin typing. The procedures followed were in accord with the ethical standards established by the institution at which the experiments were performed or were in accord with the Helsinki Declaration of 1975.

### Hematological Parameters and Erythropoietin Level

Blood cells and red cell indices were analyzed with a Coulter counter (model ZX6). Hemoglobin typing was performed by automated high-performance liquid chromatography (Bio-Rad). EPO level was measured by enzyme-linked immunosorbent assay (ELISA).

### Erythroid Progenitor Cell Culture and TNF-a Treatment

CD34-positive cells (105 cells/mL) were isolated from peripheral blood mononuclear cells using the EasySep® CD34 selection kit, following the manufacturer’s instructions, and were cultured in Iscove’s modified Dulbecco’s medium (GIBCO) supplemented with 15% human AB serum, 15% fetal calf serum in the presence of 10 ng/mL recombinant interleukin-3, 20 ng/mL stem cell factor, and various concentrations of EPO (0, 0.2, 2, and 20 U/mL). For TNF-a treatment, cells were incubated with 20 ng/mL of TNF-α and incubated at 37 °C in 5% CO2 for 14 days. CD34-positive cells were checked by flow cytometry and erythroid progenitor cell development was observed by Wright-Giemsa staining.

### Total Cell and Viability Assay by Trypan Blue Staining

Trypan blue solution was used for cell viability assay. To determine total cell count and cell viability, 20 µL of cell suspension was mixed with 20 µL of 0.4% trypan blue solution. Viable cells and number of total cells were counted by hemocytometer.

### Detection of Percent Apoptosis of Erythroid Progenitor Cells

Apoptosis was assessed by flow cytometry according to the manufacturer’s protocol. First, erythroid cultured cells were washed with 1 mL of cold D-PBS. After centrifugation at 12,000 rpm for 5 min, 100 µL of room-temperature 1X Annexin V binding buffer was added to the pellet. Next, 2 µL of Annexin V-FITC and 5 µL of glycophorin A-PE antibody were mixed into the cell suspension; this mixture was incubated for 15 min in the dark and then 100 µL of 1X Annexin V binding buffer was again mixed into the cell suspension. Finally, the cells were analyzed using a FAC Sort flow cytometer (BD Biosciences, USA). At least 10,000 cells were counted in order to determine the percentage of apoptosis.

### Measuring Erythropoietin Receptor Protein by Flow Cytometry

Erythroid progenitor cells were cultured for 14 days. Cells were then incubated with anti-EPOR labeled with FITC and the percentage of EPOR protein was measured by flow cytometry.

### Statistical Analysis

Results are expressed as mean ± SD. Statistical analysis was performed using a nonparametric Kolmogorov-Smirnov test test and Student’s t-test. Significance was set at p<0.05.

## RESULTS

### Hematological Data and Level of Erythropoietin from Healthy Subjects and β-Thalassemia/Hemoglobin E Patients

**Figure 1:**
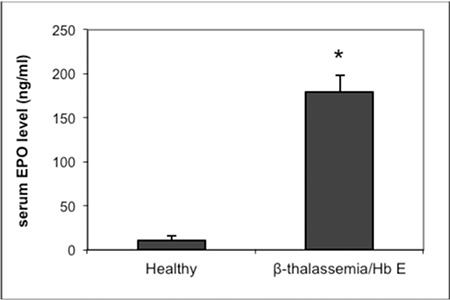
Serum erythropoietin levels of healthy control subjects and beta thalassemia/hemoglobin E patients. *: p<0.05 compared with healthy subjects.

Hematological parameters are summarized in Table 1. Serum EPO level was measured by ELISA and is shown in Figure 1. The level of serum EPO in β-thalassemia/Hb E cases was statistically significant higher than in healthy subjects.

### Role of Erythropoietin on Cell Proliferation of Erythroid Progenitor Cells

**Figure 2:**
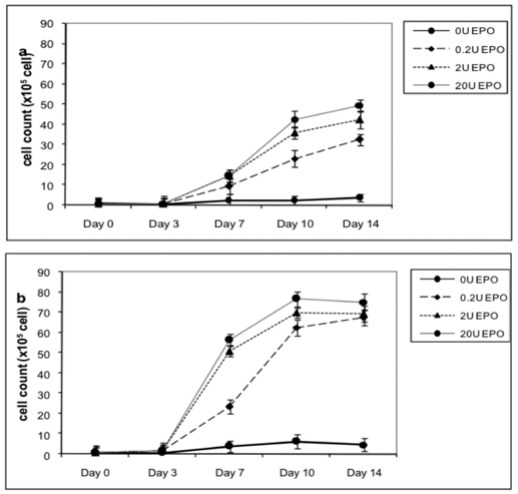
Cell count of erythroid progenitor cells from healthy control subjects a) and β-thalassemia/hemoglobin E patients b) after treatment with various concentrations of erythropoietin for 14 days.

The effect of EPO on cell proliferation of erythroid progenitor cells was investigated by trypan blue staining. The results suggested that EPO increased the number of erythroid progenitor cells in a dose- and time-dependent manner. In addition, thalassemic patients had higher cell numbers than healthy subjects (Figure 2).

### TNF-α Inhibits Erythropoietin-Induced Erythroid Cell Proliferation

**Figure 3:**
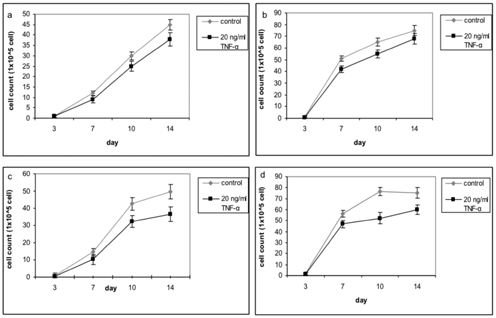
Effect of TNF-a on cell count of erythroid progenitor cells treated with 2 U EPO (a) and 20 U (c) of healthy and 2 U (b) and 20 U (d) of b-thalassemia/Hb E.

The effect of TNF-α on EPO-induced erythroid progenitor cell proliferation was studied. TNF-a caused a reduction of erythroid progenitor cells in both groups; however, this effect was stronger among β-thalassemia/Hb E patients (Figure 3).

### Role of Erythropoietin on Apoptosis of Erythroid Progenitor Cells

**Figure 4:**
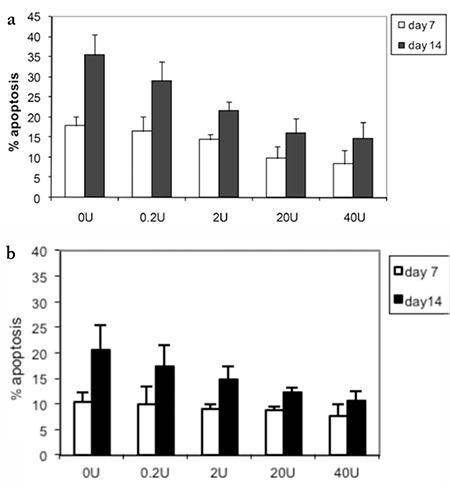
Percent cell apoptosis of erythroid progenitor cells from healthy control subjects a) and β-thalassemia/hemoglobin E patients b) after treatment with various concentrations of erythropoietin for 14 days as analyzed by flow cytometry. *: p<0.05 compared with day 7.

Various concentrations of EPO were added to erythroid progenitor cells and percent cell apoptosis was analyzed by flow cytometry. EPO caused a reduction of percent apoptosis in erythroid progenitor cells in a dose-dependent manner. β-Thalassemia/Hb E cells had a higher percentage of apoptosis than the cells of healthy subjects (Figure 4).

### TNF-a Induced Apoptosis in Erythropoietin-Treated Cells

**Figure 5:**
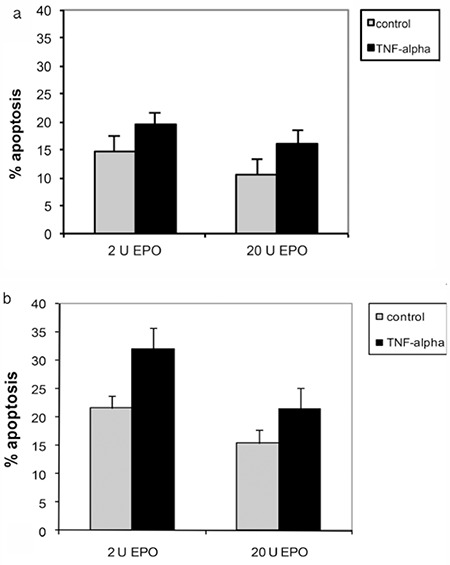
Effect of tumor necrosis factor-alpha on percent cell apoptosis of erythroid progenitor cells from healthy control subjects (a) and β-thalassemia/hemoglobin E patients (b) after treatment with 2 U and 20 U erythropoietin for 14 days as analyzed by flow cytometry. *: p<0.05 compared with the control.

The effect of TNF-a on induction of apoptosis of erythroid progenitor cells treated with EPO showed that percent apoptosis of TNF-a-treated cells was statistically significant higher than in the control in cells treated with both 2 U/mL and 20 U/mL EPO (Figure 5).

### Role of Erythropoietin on Erythropoietin Receptor Protein of Erythroid Progenitor Cells

**Figure 6:**
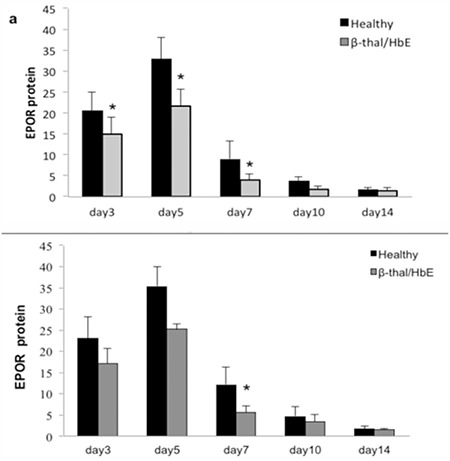
Erythropoietin receptor protein of erythroid progenitor cells from healthy control subjects and β-thalassemia/hemoglobin E patients after treatment with 2 U (a) and 20 U (b) erythropoietin for various times as measured by flow cytometry. *: p<0.05 compared with the healthy control.

The level of EPOR protein was measured by flow cytometry and the results showed that the level of EPOR in erythroid progenitor cells from β-thalassemia/Hb E patients was lower than in those from healthy subjects. The highest EPOR protein level was shown in EPO-treated erythroid cells from healthy subjects at day 5 of culture (Figure 6).

### TNF-α Inhibits Erythropoietin Receptor Protein of Erythroid Progenitor Cells

**Figure 7:**
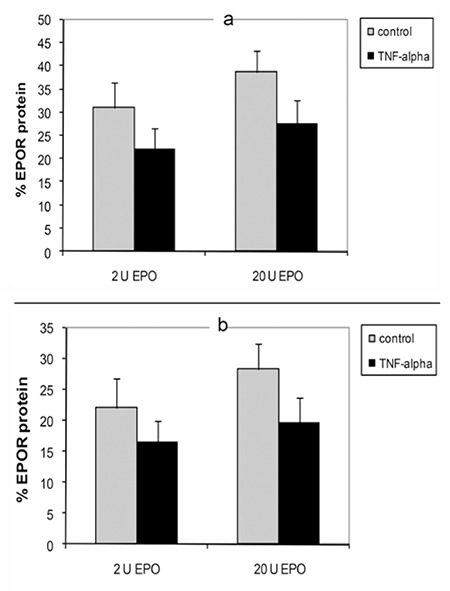
Figure 7. Effect of tumor necrosis factor-alpha on erythropoietin receptor protein levels of erythroid progenitor cells from healthy control subjects (a) and β-thalassemia/hemoglobin E patients (b) after treatment with 2 U and 20 U on day 5 of culture as measured by flow cytometry. *: p<0.05 compared with the control.

After adding TNF-a to erythroid progenitor cells treated with EPO, lower levels of EPOR protein were seen in erythroid progenitor cells from both healthy subjects and β-thalassemia/Hb E patients (Figure 7).

## DISCUSSION

β-Thalassemia/Hb E is a thalassemic syndrome that results from co-inheritance of the hemoglobin E trait with either β0 or β+ thalassemia. The severity of the disease is very variable, ranging from minor through intermediate to major. Many studies have tried to explain the severity based on pathophysiological factors such as ineffective erythropoiesis. Ineffective erythropoiesis is characterized by apoptosis of the erythroid progenitor cells [6]. Many proteins have the potential to affect erythroid proliferation and differentiation. Interestingly, the level of serum EPO in β-thalassemia/Hb E patients was higher than normal. A previous study reported that cells become progressively more sensitive to EPO during erythroid differentiation due to the appearance of EPOR [7]. In this study, the highest EPOR protein levels were seen at day 5 of culture in erythroid progenitor cells from healthy subjects; the majority of cells were pronormoblasts. In addition, EPOR protein levels in thalassemic patients were lower than in healthy subjects. The level of EPOR might be associated with the stage of erythroid cells. There are reports on the relation of EPO and EPOR expression in other cells, such as endothelial cells and head and neck squamous cell carcinoma [8]. In this study, a reduction of percent cell apoptosis was found in EPO-treated cells. The percent apoptosis of thalassemic patients was higher than that of healthy subjects, which might be related to ineffective erythropoiesis in β-thalassemia/Hb E patients.

Recent studies reported that cytokines could be involved with ineffective erythropoiesis in β-thalassemia. A previous study by our group showed that cytokines, including TNF-α and interferon-γ, had the potential to induce nitric oxide, involved with apoptosis of erythroid progenitor cells from β-thalassemia/Hb E patients [9]. However, the mechanism of TNF-α involved in EPO regulation remains unclear. TNF-α is one of the proinflammatory cytokines that reportedly inhibit generation of glycophorin A+ cells [10], and decreased differentiation of erythroid cells exacerbates ineffective erythropoiesis in b-thalassemia [11]. In addition, the serum level of TNF-α was statistical significantly higher in postsplenectomized thalassemic patients than in normal controls and nonsplenectomized patients, which indicated that TNF-α could play a role in the pathogenesis of the disease [12]. One previous study reported that the TNF-α levels of b-thalassemia/Hb E patients were higher than normal in only 13% of the patients [13]. However, many studies have shown an increased TNF-α concentration in b-thalassemia major patients [12,14]. It was suggested that the increase in TNF-α could be caused by macrophage activation due to iron overload and the antigenic stimulation induced by chronic transfusion therapy. The activated macrophages were selectively phagocytosing apoptotic erythroid precursors, thereby contributing to ineffective erythropoiesis [15]. In this study it was demonstrated that TNF-α caused higher levels of apoptosis in b-thalassemia/Hb E erythroid progenitor cells compared to cells from the control group. In addition, EPOR protein in erythroid progenitor cells was inhibited by this cytokine. This suggests that TNF-α caused a reduction of both EPOR protein expression and EPO-induced cell proliferation of thalassemic erythroid progenitor cells, which could be involved in the mechanism of ineffective erythropoiesis in b-thalassemia/Hb E patients.

## ACKNOWLEDGMENTS

This work was supported by the Thailand Research Fund (Grant No. MRG5180127) and by a Mahidol University Research Grant.

**Ethics Committee Approval:** COA No. MU-IRB 2009/252.2910, **Informed Consent:** It was taken, **Concept:** Dalina I Tanyong, Prapaporn Panichob, Wasinee Kheansaard, Suthat Fucharoen, **Design:** Dalina I Tanyong, Prapaporn Panichob, Wasinee Kheansaard, Suthat Fucharoen, **Data Collection or Processing:** Dalina I Tanyong, Prapaporn Panichob, Wasinee Kheansaard, Suthat Fucharoen, **Analysis or Interpretation:** Dalina I Tanyong, Prapaporn Panichob, Wasinee Kheansaard, Suthat Fucharoen, **Literature Search:** Dalina I Tanyong, Prapaporn Panichob, Wasinee Kheansaard, Suthat Fucharoen, **Writing:** Dalina I Tanyong, Prapaporn Panichob, Wasinee Kheansaard, Suthat Fucharoen.

**Conflict of Interest:** The authors of this paper have no conflicts of interest, including specific financial interests, relationships, and/or affiliations relevant to the subject matter or materials included.

## Figures and Tables

**Table 1:**
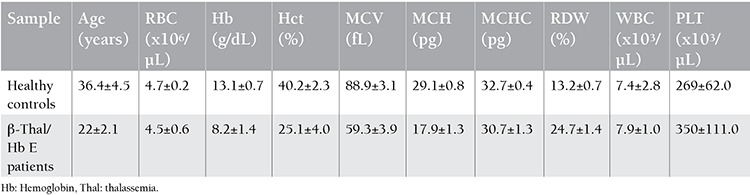
Hematological parameters of healthy subjects and beta-thalassemia/hemoglobin E patients.
